# The Background and Rationale Behind the Establishment of the First AIDS and the Elderly GSA Interest Group

**DOI:** 10.1093/geroni/igaa057.3111

**Published:** 2020-12-16

**Authors:** Lynn Tepper

**Affiliations:** CDM, New York City, New York, United States

## Abstract

Although HIV became known during the decade of the 1980’s, it was not until 1987 that the WHO launched the Global Program on AIDS to raise awareness, generate evidence-based policies, and provide technical and financial support to conduct research, promote NGO participation, and promote the rights of people living with HIV. It was then also that robust educational and prevention initiatives began to take place. At this time, a Columbia University study, led by Dr. Lynn Tepper, a gerontologist, initiated a study to see if the older population fully understood the disease and the practice of prevention behaviors, as they were not a specific target for AIDS education and prevention. This led to the establishment of the AIDS and the Elderly Interest Group, sponsored by GSA, in an attempt to know more about the impact of AIDS in the older population. Part of a symposium sponsored by the HIV, AIDS and Older Adults Interest Group.

